# Comparative pulmonary toxicity of a DWCNT and MWCNT-7 in rats

**DOI:** 10.1007/s00204-018-2336-3

**Published:** 2018-10-19

**Authors:** Ahmed M. El-Gazzar, Mohamed Abdelgied, David B. Alexander, William T. Alexander, Takamasa Numano, Masaaki Iigo, Aya Naiki, Satoru Takahashi, Hiroshi Takase, Akihiko Hirose, Jun Kannno, Osama Saeid Elokle, Ashraf Mohamed Nazem, Hiroyuki Tsuda

**Affiliations:** 1 Nanotoxicology Project, Nagoya City University, Nagoya, Japan; 2 Department of Experimental Pathology and Tumor Biology, Graduate School of Medical Sciences, Nagoya City University, Nagoya, Japan; 3 Core Laboratory, Graduate School of Medical Sciences, Nagoya City University, Nagoya, Japan; 4 Division of Risk Assessment, National Institute of Hygienic Sciences, Kawasaki, Japan; 5 Bioassay Research Center, Japan Industrial Safety and Health Association, Kanagawa, Japan; 6 Department of Forensic Medicine and Toxicology, Faculty of Veterinary Medicine, Alexandria University, Alexandria, Egypt; 7 Department of Forensic Medicine and Toxicology, Faculty of Veterinary Medicine, Beni Suef University, Beni Suef, Egypt; 8 Department of Food Hygiene, Faculty of Veterinary Medicine, Alexandria University, Alexandria, Egypt

**Keywords:** Double-walled carbon nanotubes, DWCNT, Inhalation toxicity, Rat, Intra-tracheal intra-pulmonary spraying, TIPS

## Abstract

Very little is known about the in vivo toxicity of inhaled double-walled carbon nanotubes (DWCNTs). In the present study, we compared the pulmonary toxicity of DWCNT to MWCNT-7, a well-known multi-walled carbon nanotube. Rats were divided into six groups: untreated, vehicle, low-dose DWCNT, high-dose DWCNT, low-dose MWCNT-7, and high-dose MWCNT-7. The test materials were administered by intra-tracheal intra-pulmonary spraying (TIPS) every other day for 15 days: the low-dose and high-dose groups were administered final total doses of 0.25 and 0.50 mg/rat of the test material. The animals were sacrificed 1 and 6 weeks after the final TIPS administration. Six weeks after the final TIPS administration, rats administered MWCNT-7 had high levels of macrophage infiltration into the lung with dense alveolar wall fibrous thickening throughout the lung; significant elevation of lactate dehydrogenase activity, alkaline phosphatase activity, and total protein concentration in the bronchioalveolar lavage fluid; an increase in the pulmonary cell PCNA index; slightly elevated levels of 8-OHdG DNA adducts in lung tissue DNA; a small but significant increase in protein concentration in the pleural cavity lavage fluid and an increase in the visceral mesothelial cell PCNA index. None of these parameters was increased in rats administered DWCNT. The primary lesion in rats administered DWCNT was scattered formation of granulation tissue containing internalized DWCNT fibers. Our data indicate that DWCNT has lower pulmonary and pleural toxicity than MWCNT-7.

**Electronic supplementary material:**

The online version of this article (10.1007/s00204-018-2336-3) contains supplementary material, which is available to authorized users.

## Introduction

Carbon nanotubes (CNTs) are composed of one or more one-atom thick graphene cylinders. The carbon–carbon bonds of graphene are exclusively *sp*^2^, which gives CNTs remarkable mechanical, thermal, chemical, and electrical properties, and their hollow tubular structure makes them very light weight. However, these properties also make CNTs easily airborne and highly biopersistent in tissues when inhaled. Consequently, the possibility that CNTs may exhibit foreign body toxicity is of great concern. Notably, two multi-walled CNTs, MWCNT-7 and MWCNT-N, have been shown to be carcinogenic in rats after whole-body inhalation exposure (MWCNT-7; Kasai et al. 2016) or administration by intra-tracheal intra-pulmonary spraying (TIPS) (MWCNT-N; Suzui et al. 2016): in Kasai et al. (2016), the term MWNT-7 is used to refer to the MITSUI MWCNT-7 used in their study.

Currently, very little is known about the in vivo toxicity of inhaled double-walled carbon nanotubes (DWCNTs). Crouzier et al. (2010) administered 1.5 mg/kg DWCNT (1.2–3.2 nm diameter, 1–10 µm length) to male Swiss mice by intranasal instillation and examined the mice 6 h, 24 h, and 48 h after instillation. They report that DWCNT administration caused an inflammatory reaction but that the inflammatory reaction was accompanied by a decrease in reactive oxygen species production in the lung. Tian et al. administered 50 µg DWCNT (3.5 nm diameter, 1–10 µm length) to female BALB/cAnNCr mice by intratracheal instillation and examined the mice at 1 day, 3 days, and 7 days after instillation (Tian et al. 2013). They report that DWCNT administration caused pulmonary inflammation that persisted for 7 days. Sager et al. administered up to 40 µg DWCNT (1–2 nm diameter, < 5 µm length: manufactured by Toray) to male C57BL/J mice by pharyngeal aspiration (Sager et al. 2013). They report that 56 days after administration, 40 µg DWCNT induced a greater degree of alveolitis and lung fibrosis than a similarly administered MWCNT. O’Shaughnessy et al. (2014) administered DWCNT (< 5 nm diameter, 5–15 µm length) to mice by whole-body inhalation: 10.8 mg/m^3^, 4 h/day for 5 days. They report that immediately after exposure DWCNT caused inflammation and tissue injury in the lung, but that 2 weeks after the last exposure these pathological changes had resolved.

The present report is part of a study undertaken to assess the long-term effects of a DWCNT, and to compare the effects of this DWCNT with MWCNT-7, a known carcinogen in rats. Rats were administered 250 µg or 500 µg CNTs by intra-tracheal intra-pulmonary spraying (TIPS) and sacrificed 1 week and 6 weeks after the final TIPS administration. Rats administered DWCNT developed scattered formation of granulation tissue, but tissue damage parameters in the bronchioalveolar lavage and pleural lavage fluids, PCNA indices in the lung and pleura, and 8-OHdG adduct levels in the lung tissue were not changed compared to the control groups. In contrast, rats administered MWCNT-7 had extensive inflammatory lesions, increases in tissue damage parameters in the lung and pleura, increases in the PCNA indices in the lung and pleura, and increased 8-OHdG levels in lung tissue DNA. Overall, these preliminary results suggest that the DWCNT we are examining is less toxic than MWCNT-7. Our ongoing 2-year study will resolve the toxicity of this DWCNT in the rat lung and determine the relevance of the findings reported here to its long-term toxicity.

## Materials and methods

### Materials

We used two types of carbon nanotubes in this study, DWCNT (brand name Tocana; Toray Industries, Inc., Tokyo, Japan) with an iron content below detectable limits (information provided by the company) and MWCNT-7 (Mitsui Chemicals Inc., Tokyo, Japan) with an iron content of 3500 ppm (Takagi et al. 2008, 2012).

### Animals

Nine-week old male F344/DuCrlCrlj rats were purchased from Charles River Japan Inc. (Kanagawa, Japan). The animals were housed in the center for experimental animal science of Nagoya City University Medical School, maintained on a 12 h light–dark cycle, and received Oriental MF basal diet (Oriental Yeast Co., Tokyo, Japan) and water ad libitum. The experimental protocol was approved by the Animal Care and Use Committee of Nagoya City University Medical School, and the research was conducted according to the Guidelines for the Care and Use of Laboratory Animals of Nagoya City University Medical School. The experiment was started after a 2-week acclimation and quarantine period.

## Preparation of the test substance suspension

The two test materials were weighed and then dispersed in tert-butyl alcohol by sonication for 10 min and stored frozen at − 40 °C as per Taquahashi et al. (2013). Shortly before administration, the *T*-butyl alcohol was removed using an Eyela Freeze Drying machine (FDU-2110; Tokyo Rikakikai Co., Ltd., Tokyo, Japan), and DWCNT and MWCNT-7 were suspended in saline containing 0.5% Pluronic F-68 (PF68, Sigma-Aldrich, St. Louis, MO, USA) at 62.5 and 125 µg/ml. After suspension in Saline + PF68, test materials were sonicated for 2 min four times at 3000 rpm using a polytron PT 1600E bench top homogenizer (Kinematika AG, Lattau, Switzerland). Immediately prior to administration, the suspensions were sonicated for 30 min using a Tomy Ultrasonic disruptor, UD-211, equipped with a TP-040 micro tip (Tomy Seiko Co., Ltd., Tokyo, Japan) at a power setting of 4.

## Characterization of the test substance in suspension

After sonication, 20 µl of each test material suspension was placed on a micro grid membrane pasting copper mesh (EMS 200-Cu, Nisshin EM Co., Ltd., Tokyo, Japan); the shape of the nanoparticles was imaged by transmission electron microscopy (JEOL Co. Ltd, Tokyo, Japan), and the photos were analyzed by NIH image analyzer software (NIH, Bethesda, Maryland, USA). Over 1000 fibers of each type of CNT were measured.

## Experimental design

A total of 96 rats 11 weeks old were divided into six groups of 16 animals each: Group 1, without treatment; Group 2, vehicle (PF 68 + saline); Group 3, DWCNT (0.25 mg); Group 4, DWCNT (0.50 mg); Group 5, MWCNT-7 (0.25 mg); and Group 6, MWCNT-7 (0.50 mg). Rats under 3% isoflurane anesthesia were administered 0.5 ml vehicle or test material suspensions (31.25 or 62.5 µg of test material in 0.5 ml vehicle) by intra-tracheal intra-pulmonary spraying (TIPS) using a microsprayer (series IA–1B Intratracheal Aerosolizer Penn-century, Philadelphia, PA) as described previously (Xu et al. 2010, 2012). Rats were treated once every other day over a 15-day period (8 times for total doses of 0.25 or 0.5 mg test material/rat). The amount of DWCNT administered to the rats was approximately equivalent to the doses used in the studies by Crouzier et al. (2010), Tian et al. (2013), and Sager et al. (2013), discussed in the “Introduction”. The corresponding dose of MWCNT-7 was half the amount of MWCNT-N that caused lung cancer and mesothelioma in rats (Suzui et al. 2016). One and six weeks after the final TIPS administration, eight rats from each group were sacrificed by exsanguination from the abdominal aorta under deep anesthesia. At 1 week after the final TIPS administration, one of the measured parameters (macrophage counts) was elevated in the vehicle control group compared to the untreated group (see Supplementary Material Table 3). Therefore, only the data obtained at experimental week 8 was used to assess the toxicity of DWCNT.

## Tissue sample collection

At 1 week after the final TIPS administration, the lung was excised and the right superior, right middle, and right inferior lobes were divided into two parts and immediately frozen at − 80 °C, and the left lung was fixed in 4% paraformaldehyde solution adjusted to pH 7.3 and processed for haematoxylin–eosine (H&E) staining and immunohistochemical examination. At 6 weeks after the final TIPS administration, the left lung was used for collection of bronchioalveolar lavage fluid and the right superior, right middle, and right inferior lobes were divided into two parts; the first part was immediately frozen at − 80 °C, and the second part as well as the trachea and mediastinal lymph node were fixed in buffered 4% paraformaldehyde solution and processed for haematoxylin–eosine (H&E) staining and immunohistochemical examination. Liver, kidney, spleen, heart, and brain were collected and processed for haematoxylin–eosine (H&E) staining.

### Light microscopy, polarized light microscopy, and electron microscopy

Identification of the administered CNTs in the tissue and alveolar macrophages was confirmed by polarized light microscopy (PLM, Olympus, Tokyo, Japan). For high magnification viewing, slides were immersed in xylene to remove the cover glass, then immersed in 100% ethanol, air-dried and coated with platinum, and viewed by SEM (Field Emission Scanning Electronic Microscope; Hitachi High Technologies, Tokyo, Japan) at 5–10 kV.

### Immunohistochemical analysis

PCNA staining of lung sections was performed as previously described (Abdelgied et al. 2018). In each lung specimen more than 1000 pulmonary epithelial cells and more than 500 visceral pleural mesothelial cells were counted blindly in random fields. All nuclei showing brown staining of more than half of the nucleus were considered to be positive.

For CD68 staining (for macrophage counting), paraffin-embedded sections were incubated with PBS containing 5% BSA and 5% goat serum for 1 h, then incubated with anti-CD68 (BMA Biomedicals, Augst, Switzerland) diluted 1:2000 in PBS containing 1% BSA and 1% goat serum overnight at 4 °C. After overnight incubation, the slides were incubated with secondary antibody (Nichirei Biosciences, Tokyo, Japan) for 1 h, visualized with DAB (Nichirei Biosciences, Tokyo, Japan), and counterstained with hematoxylin. The number of macrophages was counted in 20 random fields from each lung section.

### Collection of bronchioalveolar lavage fluid (BALF), pleural lavage fluid (PLF), and preparation of the pleural cavity lavage cell pellet

Collection of the BALF and PLF and preparation of the pleural cavity lavage cell pellet was performed as previously described (Abdelgied et al. 2018).

### Measurement of lactate dehydrogenase (LDH), alkaline phosphatase (ALP), and total protein

Lactate dehydrogenase (LDH) activity was measured using an LDH activity assay kit (MAK066: Sigma-Aldrich, St Louis, MO, USA). Alkaline phosphatase (ALP) activity was measured using an ALP enzyme activity assay kit (291-58601: Wako Chemicals Co., Ltd., Tokyo, Japan). Total protein concentration was measured using the BCA Protein assay kit (23227: Pierce biotech, Rockford, IL, USA).

### mRNA isolation, cDNA synthesis, and RT-PCR analysis of gene expression

RNA isolation was performed as previously described (Abdelgied et al. 2018). The RNA integrity numbers of all samples were higher than seven. Reverse transcription of the isolated RNA was performed as previously described (Abdelgied et al. 2018). Primers were designed using Primer Premier 6.11 software (Premier Biosoft International, CA, USA) and purchased from Sigma-Aldrich. Primers are listed in Supplementary Material Table 1.

## Measurement of cytokine levels by ELISA

Frozen right lung tissue samples (approximately 100 mg) were thawed and rinsed three times with ice cold PBS and homogenized in 1 ml of tissue protein extraction reagent (Thermo Scientific, Rockford, Illinois, USA) containing 1% (v/v) protease inhibitor cocktail (Sigma-Aldrich, St Louis, MO, USA). The homogenates were centrifuged at 12,000x*g* for 5 min at 4 °C. The protein content of the supernatant was measured using the BCA Protein assay kit (Pierce Biotech, Rockford, IL, USA). The levels of CCL2, CCL3, and CCL4 in the supernatant was measured using Rat CCL2 (LS-F37066), CCL3 (LS-F5526), and CCL4 (LS-F5525) ELISA Kits from LSBio (Seattle, Washington, USA) according to the manufacturer’s instructions.

## Analysis of 8-hydroxydeoxy guanosine levels (8-OHdG)

Genomic DNA was extracted from a piece of the right lung using a Qiagen DNeasy blood and tissue kit (Qiagen, Germany). The extracted DNA was digested with DNase I [Takara Biotechnology (Dalian) Co., Japan] and Nuclease P1 (Sigma N8630) according to the manufacturers’ instructions (Huang et al. 2001). The digested DNA was incubated with alkaline phosphatase (Sigma P 5931) at 1 unit AP/100 µg DNA at 37 °C for 30 min, and boiled for 10 min and placed on ice. The 8-OHdG levels were measured with an 8-OHdG ELIZA kit (ab201734; abcam, Tokyo, Japan).

### Statistical analysis

All data are expressed as mean values ± standard deviation. Data were analyzed for homogeneity of variance using Levene’s test. Statistical significance was analyzed by one-way ANOVA when the variance was homogenous and Welch’s *t* test when the variance was not homogenous using SPSS software (IBM, Armonk, New York, USA). *P* values less than 0.05 were considered to be statistically significant.

## Results

### Characterization of DWCNT and MWCNT-7 in suspension

**Fig. 1:**
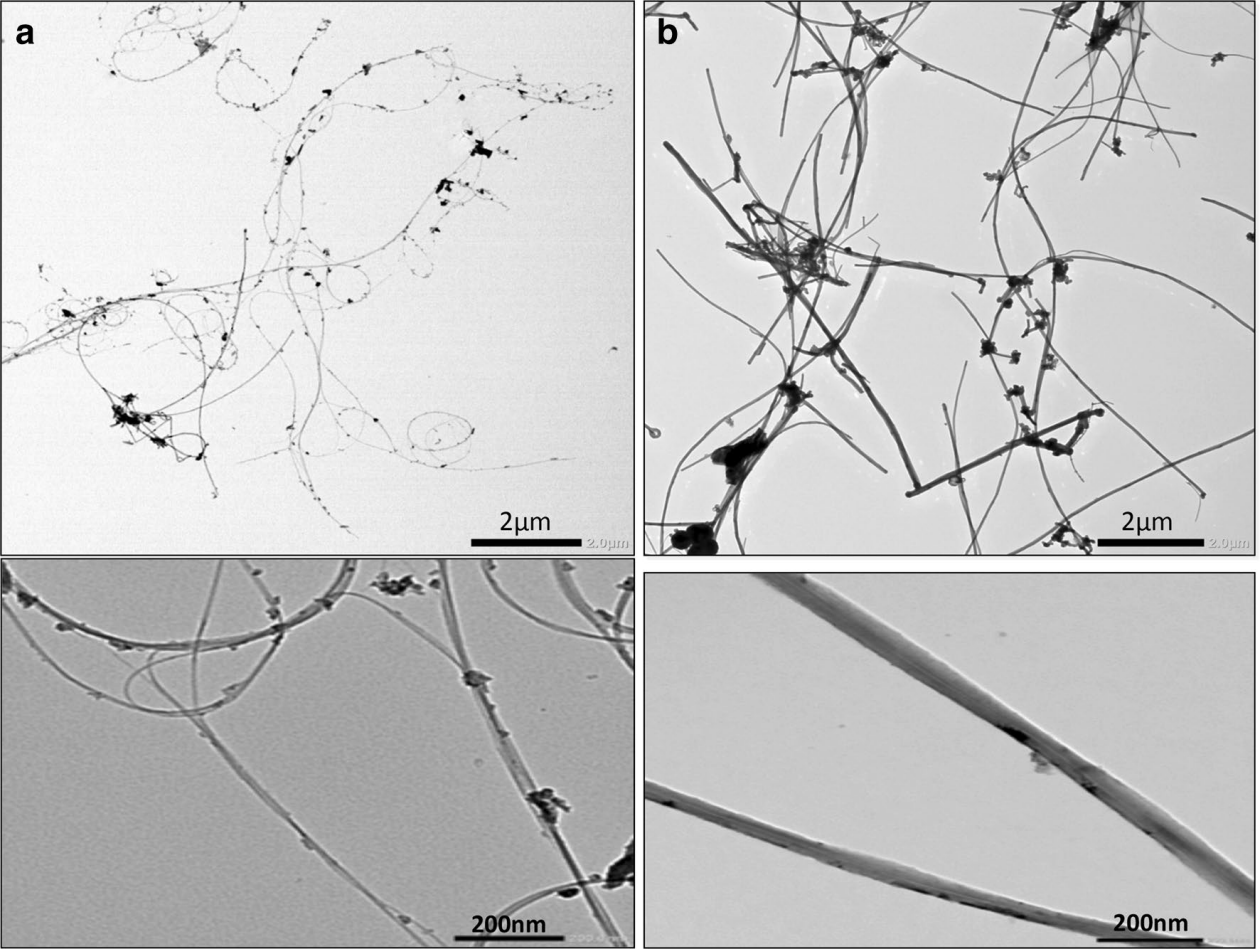
TEM images of DWCNT (**a**) and MWCNT-7 (**b**) in suspension. The photos in the lower panels show higher magnification images of DWCNT (**a**) and MWCNT-7 (**b**)

TEM images of DWCNT and MWCNT-7 are shown in Fig. 1. DWCNT fibers are tangled with a mean diameter of 1–3 nm. The length could not be measured due to the tangled nature of the fibers. Individual MWCNT-7 fibers are needle-like. The mean diameter of MWCNT-7 was 55.5 ± 12 nm and the mean length was 6.5 ± 2.4 µm with a median length of 6.62 µm.

### Pathological findings (week 8)

**Fig. 2:**
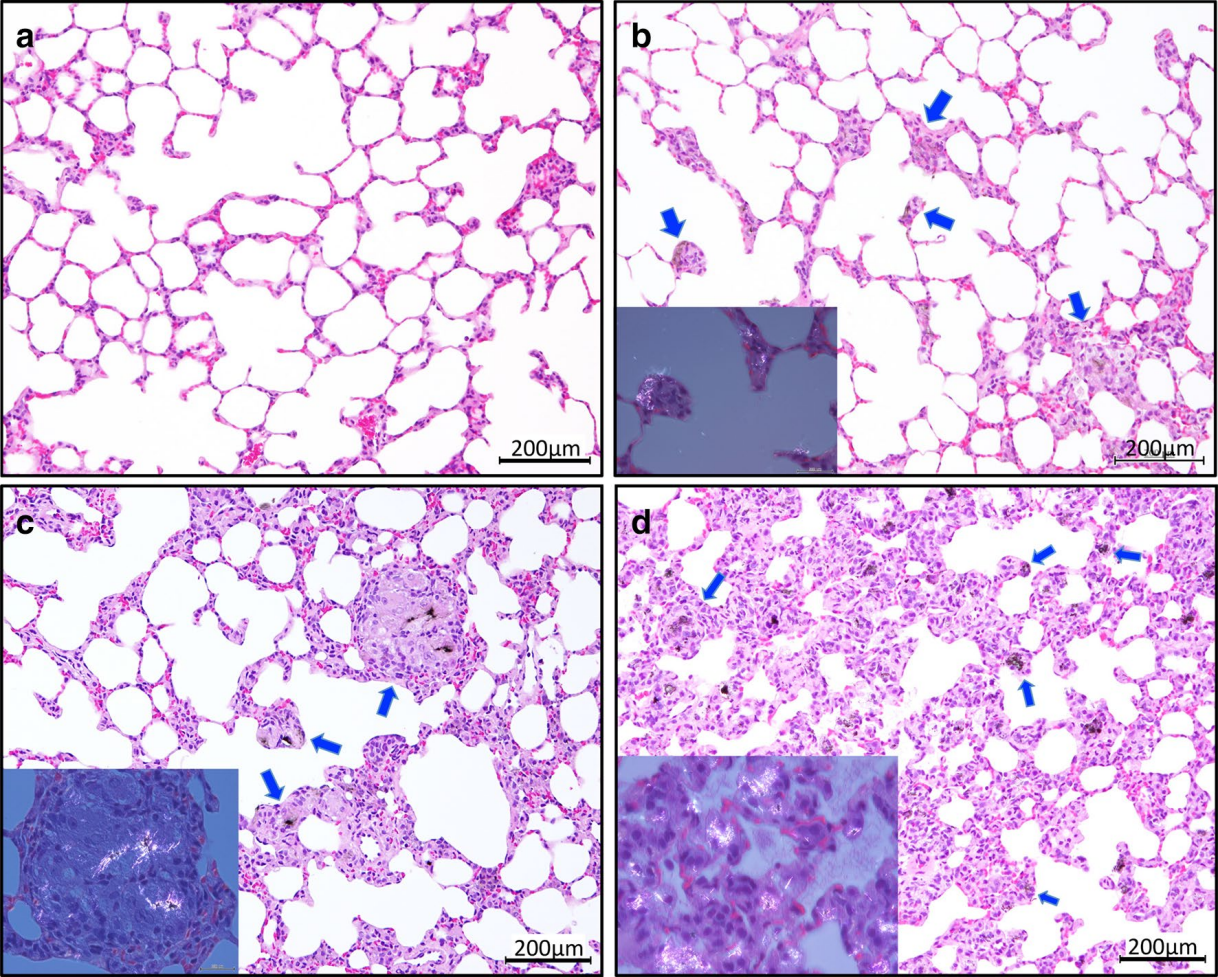
Typical sections of lung tissue at experimental week 8 from rats administered saline + PF-68 (**a**), DWCNT (0.25 mg) (**b**), DWCNT (0.5 mg) (**c**), and MWCNT-7 (0.5 mg) (**d**). Arrows indicate granulation tissue internalizing DWCNT and MWCNT-7 fibers. Insets show polarizing lens views (color figure online)

Typical lung sections from rats administered vehicle, DWCNT, or MWCNT-7 are shown in Fig. 2. Inflammatory changes in rats administered 0.25 mg DWCNT was milder than in rats administered 0.50 mg DWCNT. Inflammatory changes in rats administered MWCNT-7 was stronger than in rats administered DWCNT. Sporadic mottled thickening of the alveolar wall with early-stage granulation tissue was seen in in the lungs of rats administered both low and high doses of DWCNT. In rats administered MWCNT-7, granulation tissue and dense alveolar wall fibrous thickening were found throughout the lung tissue.

**Fig. 3:**
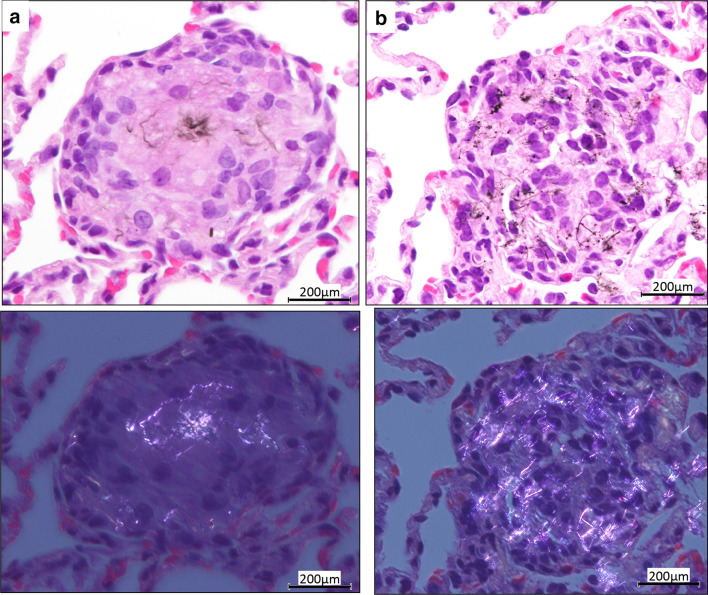
A granulation lesion from a rat administered DWCNT (**a**) and MWCNT-7 (**b**) at experimental week 8. The lower photos are polarizing lens images showing the presence of DWCNT and MWCNT-7 fibers in the granulation tissue (color figure online)

**Fig. 4:**
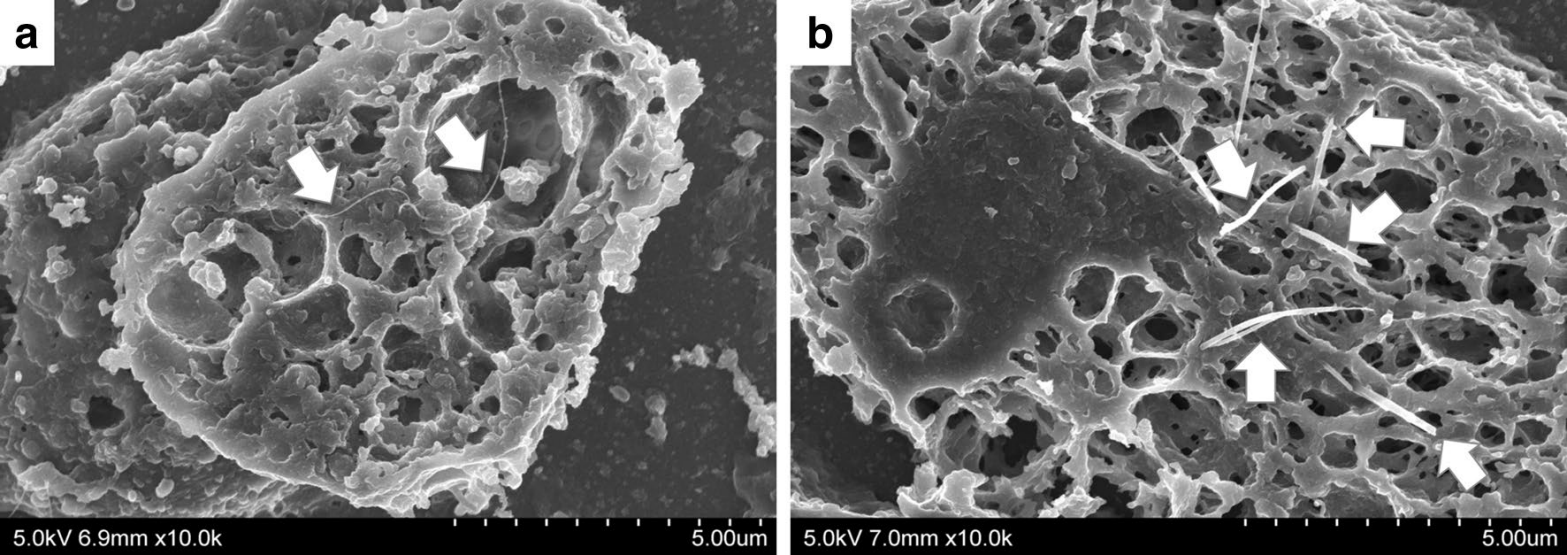
SEM images of alveolar macrophages phagocytizing DWCNT (**a**) and MWCNT-7 (**b**) (arrows) at experimental week 8

DWCNT and MWCNT-7 fibers were frequently found in multinucleated macrophages in the granulation tissue (Figs. 2, 3). In addition, in the lungs of MWCNT-7-treated rats, large numbers of free macrophages phagocytosing MWCNT-7 fibers were observed. Figure 4 shows SEM images of alveolar macrophages with DWCNT and MWCNT-7 fibers. DWCNT fibers have a thin flexible form, while MWCNT-7 fibers are long and needle-like in shape.

### Translocation of CNTs to the mediastinal lymph node and extra-pulmonary organs (week 8)

Both DWCNT and MWCNT-7 fibers were detected in the mediastinal lymph nodes (Supplementary Material Fig. 1). MWCNT-7 fibers were also detected in the pleural cavity lavage cell pellet (Supplementary Material Fig. 1) and in extra-pulmonary organs (Supplementary Material Fig. 2). DWCNT fibers could not be detected in the pleural cavity lavage cell pellet or in any organs other than the lung.

### Infiltration of alveolar macrophages (week 8)

The number of alveolar macrophages in both the low- and high-MWCNT-7 groups were significantly higher compared to the vehicle and DWCNT administered groups (*P* < 0.001) (Table 1). There was no increase in the number of alveolar macrophages in DWCNT administered rats.

**Table 1:** Lung tissue parameters at week 8

	Untreated	Vehicle^a^	DWCNT, 0.25 mg	DWCNT, 0.50 mg	MWCNT-7, 0.25 mg	MWCNT-7, 0.50 mg
Macrophage count per cm^2^ (× 10^3^)	7.58 ± 0.99	10.86 ± 1.07	9.91 ± 1.53	9.35 ± 1.07	20.26 ± 2.36***^,###^	25.94 ± 3.44***^,###^
CCL2 (pg/mg lung tissue)	114 ± 17	108 ± 22	157 ± 26*	152 ± 9*	178 ± 30***	175 ± 34***
CCL3 (pg/mg lung tissue)	108 ± 27	88 ± 27	188 ± 90*	276 ± 70^b,^***	126 ± 15	144 ± 13
CCL4 (pg/mg lung tissue)	81 ± 26	82 ± 15	127 ± 22	138 ± 25	215 ± 49***^,##^	264 ± 58***^,###^
BALF—total protein (µg/ml)	142 ± 44	156 ± 42	198 ± 37	234 ± 73	294 ± 65**	375 ± 117***^,##^
BALF—LDH (mU/ml)	0.76 ± 0.49	0.87 ± 0.53	0.72 ± 0.46	0.87 ± 0.93	5.74 ± 1.76***^,###^	7.02 ± 2.09***^,###^
BALF—ALP (U/l)	964 ± 461	1151 ± 362	1238 ± 348	1393 ± 851	3863 ± 1135***^,###^	4535 ± 1219***^,###^
8-OHdG (ng/ml lung extract)	9.24 ± 0.74	9.16 ± 0.28	9.40 ± 0.23	9.33 ± 0.22	9.94 ± 0.45**	10.21 ± 0.11***^,##^
PCNA index (%)	3.1 ± 1.5	3.3 ± 1.1	5.8 ± 2.2	5.4 ± 0.9	9.9 ± 2.1***^,###^	11.8 ± 1.6***^,###^

*,**,***Different from the vehicle control at *p* < 0.05, *p* < 0.01, *p* < 0.001, respectively

^#,##,###^Different from the respective DWCNT group at *p* < 0.05, *p* < 0.01, *p* < 0.001, respectively

^a^There were no significant differences between the untreated group and the vehicle control group

^b^There was a significant difference between the 0.25 mg DWCNT group and the 0.50 mg DWCNT group (*p* < 0.05)

### CCL2, CCL3, and CCL4, expression in lung tissue (week 8)

Expression of CCL2 and CCL3 RNA was elevated in both DWCNT and MWCNT-7-treated rats, and expression of CCL4 RNA was elevated in MWCNT-7-treated rats (Supplementary Material Table 2); therefore, protein levels of these proinflammatory cytokines were determined by ELISA (Table 1). Expression of CCL2 protein was increased in all treated groups. However, expression of CCL3 protein was elevated only in DWCNT-treated rats and expression of CCL4 protein was elevated only in MWCNT-7-treated rats. In DWCNT-treated rats, expression of CCL3 protein was significantly higher in the high-dose group compared to the low-dose group (*p* < 0.05).

### Total protein, lactate dehydrogenase (LDH) activity, and alkaline phosphatase (ALP) activity in the bronchioalveolar lavage fluid (BALF) (week 8)

Total protein was chosen an indicator of vascular permeability, and LDH and ALP were used as indicators of general cytotoxicity and type II epithelial cell toxicity, respectively (Table 1). Total protein levels were slightly, but significantly, elevated in both the low- and high-dose MWCNT-7 administered groups. LDH activity and ALP activity were both strongly elevated in the low- and high-dose MWCNT-7 administered groups. None of these parameters were elevated in either the low- or high-dose DWCNT administered groups.

### Total protein and lactate dehydrogenase (LDH) activity in the pleural lavage fluid (PLF) supernatant (week 8)

Total protein level was slightly, but significantly, elevated in the high-dose MWCNT-7 administered group (Table 2). Total protein was not elevated in the DWCNT-treated rats (data not shown). LDH levels were not elevated in either the MWCNT-7 or DWCNT-treated rats (data not shown).

**Table 2:** Pleural cavity parameters at week 8

	Untreated	Vehicle^a^	DWCNT, 0.25 mg	DWCNT, 0.50 mg	MWCNT-7, 0.25 mg	MWCNT-7, 0.50 mg
PLF—total protein (µg/ml)	727 ± 86	693 ± 33	690 ± 30	744 ± 32	747 ± 69	853 ± 117**
PLF—LDH (mU/ml)	7.70 ± 4.05	6.36 ± 3.41	6.03 ± 3.76	6.49 ± 3.04	8.54 ± 4.94	6.24 ± 1.71
PCNA index (%)	3.6 ± 1.4	6.4 ± 2.1	5.2 ± 1.2	6.1 ± 1.1	12.1 ± 3.7***^,###^	19.0 ± 2.1***^,###^

**,***Different from the vehicle control at *p* < 0.05, *p* < 0.01, *p* < 0.001, respectively

^###^Different from the respective DWCNT group at *p* < 0.001

^a^There were no significant differences between the untreated group and the vehicle control group

### 8-OHdG levels in the lung (week 8)

8-OHdG adduct formation is a marker of oxidative DNA damage and is generally associated with damage caused by reactive oxygen species (ROS). 8-OHdG levels were slightly, but significantly, increased in both the low- and high-dose MWCNT-7 treated groups (Table 1). 8-OHdG levels were not elevated in either the low- or high-dose DWCNT treated groups.

### Proliferation of pulmonary epithelial cells and visceral mesothelial cells (week 8)

**Fig. 5:**
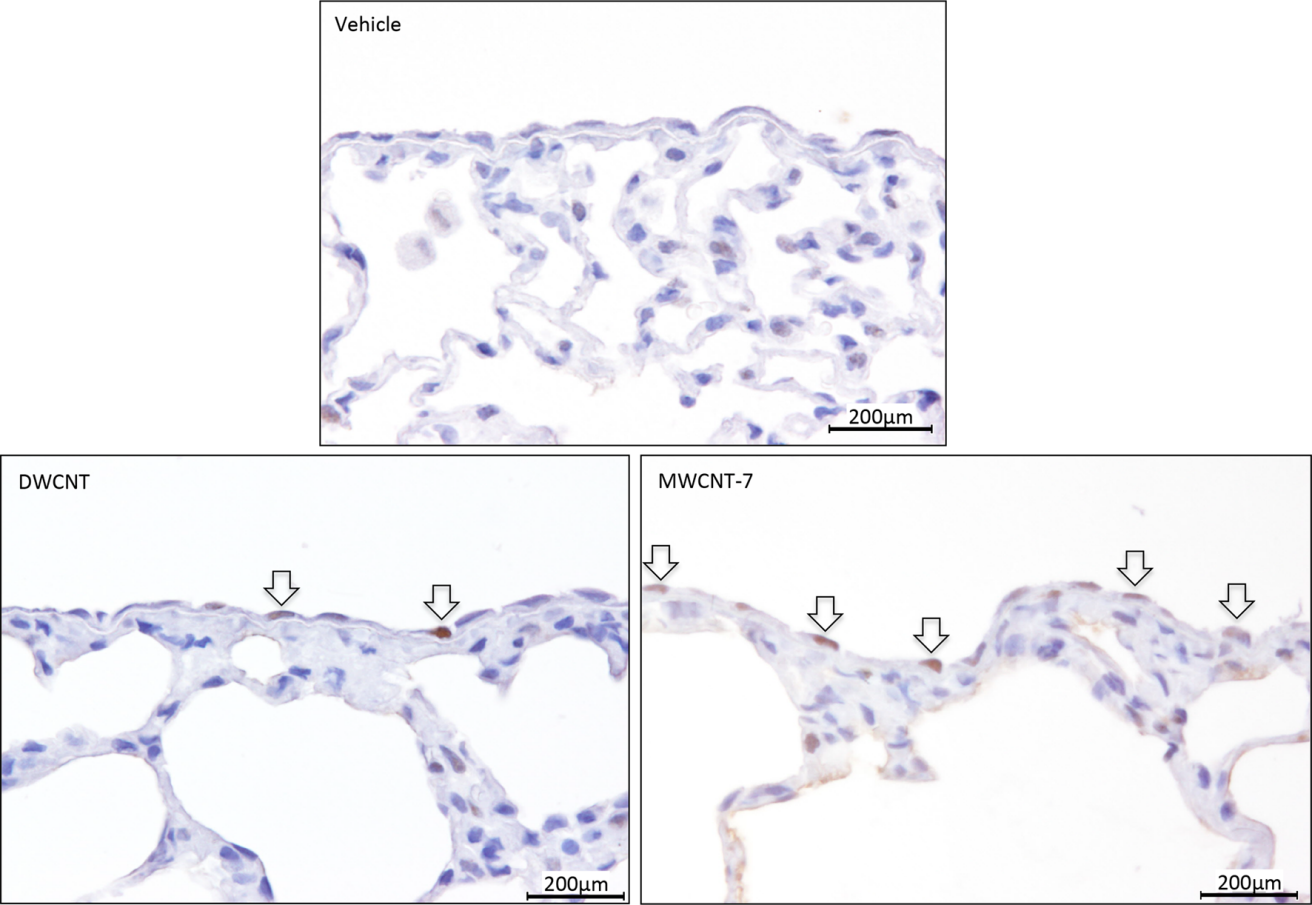
Proliferating cell nuclear antigen (PCNA) staining of visceral pleural mesothelial cells at experimental week 8. The photos show PCNA-positive cells (arrows) in the pleura of rats administered DWCNT and MWCNT-7 (color figure online)

There was a significant increase in the PCNA labeling indices of pulmonary epithelial cells in both the low- and high-dose MWCNT-7 treated groups (Table 1, Supplementary Material Fig. 3). Rats administered MWCNT-7 also had increased PCNA labeling indices in the visceral pleura (Fig. 5; Table 2). There was no significant increase in the PCNA index in either the lung or pleura in DWCNT treated rats.

## Discussion

Very little is known about the in vivo toxicity after exposure by inhalation to the various DWCNTs currently being produced. One study reported an inflammatory reaction accompanied by a decrease in reactive oxygen species production in the lung 48 h after intranasal instillation of 1.5 mg DWCNT/kg to mice (Crouzier et al. 2010). A second study reported that intratracheal instillation of 50 µg DWCNT to mice caused pulmonary inflammation that persisted for 7 days (Tian et al. 2013). A third study reported that 56 days after pharyngeal aspiration of 40 µg DWCNT to mice, DWCNT induced a greater degree of alveolitis and lung fibrosis than a similarly administered MWCNT (Sager et al. 2013). These three studies indicate that DWCNT causes inflammation in the lung. A fourth study reported that exposure of mice to 10.8 mg/m^3^ DWCNT for 4 h/day for 5 days by whole-body inhalation also caused inflammation and tissue damage in the lung, but that 2 weeks after the end of exposure the inflammation and tissue damage had resolved; this fourth study did not assess the toxicity of long-term inhalation of DWCNT (O’Shaughnessy et al. 2014). In the current study, we administered DWCNT and MWCNT-7 as a positive control reference material (Kasai et al. 2016; Warheit et al. 2007) to F344/DuCrlCrlj rats by intra-tracheal intra-pulmonary spraying (TIPS). The primary findings of the study were that 6 weeks after the final TIPS administration of MWCNT-7, parameters related to tissue damage in the lung, DNA damage in the lung, and pulmonary epithelial cell proliferation were all increased, and the total protein content of the pleural cavity lavage, which is related to tissue damage in the pleura, and cell proliferation of the visceral pleura were also increased, while in DWCNT-treated rats none of these parameters was increased. The primary lesion caused by administration of DWCNTs to the rat lung was the formation of granulation tissue with internalized DWCNT in the low-dose (0.25 mg/rat) and high-dose (0.5 mg/rat) groups.

Administration of both DWCNT and MWCNT-7 resulted in elevated levels of chemotactic cytokines in the lung tissue. In rats administered DWCNT, CCL2, and CCL3 levels were increased. This increase was likely related to the granulation tissue that was found in the lungs of these animals. Granulation tissue was also found in the lungs of rats administered MWCNT-7, as has been reported previously in MWCNT-7-exposed rats (Kasai et al. 2015, 2016; Umeda et al. 2013), and in these animals CCL2 and CCL4 levels were increased. While parameters related to tissue damage in the lung, DNA damage in the lung, and pulmonary epithelial cell proliferation were not increased in DWCNT-treated rats, the overall chemotactic cytokine levels appear to be similar in the DWCNT-exposed lung and the MWCNT-7-exposed lung (see Table 1: CCL2, CCL3, and CCL4). One possible explanation of this observation is that secretion of chemotactic cytokines may be higher in the lungs of MWCNT-7-treated rats. This possibility is supported by the observation that real-time PCR analysis indicates that RNA expression of these cytokines (Supplementary Material Table 2) is clearly higher in the MWCNT-7-exposed animals. Increased secretion can not be detected by ELISA, except as loss of tissue-associated protein; however, increased secretion of one or more of these chemotactic cytokines would account for the increase of macrophages not associated with granulation tissue, i.e., free macrophages, in the lungs of rats administered MWCNT-7 compared to the DWCNT-administered animals.

Macrophages are a major source of long rigid CNT-associated reactive oxidants (Donaldson et al. 2010, 2011; Johnston et al. 2010). The increased number of free macrophages in the lungs of MWCNT-7-treated rats (see Table 1) and their interaction with MWCNT-7 fibers (see Fig. 4b) is a likely factor in the increase of 8-OHdG adducts found in the lungs of these animals.

Total protein was used as an indication of vascular leakage, and LDH and ALP were used as indicators of general cytotoxicity and type II epithelial cell toxicity, respectively. In the lungs of MWCNT-7-treated rats, all three of these parameters were significantly increased. In contrast, none of these parameters were increased in the lungs of DWCNT-treated rats. The PCNA-index of pulmonary epithelial cells followed this toxicity pattern, indicating repair of damaged tissue in the lungs of MWCNT-7-treated rats.

Intriguingly, both DWCNT and MWCNT-7 were found in the mediastinal lymph node (see Supplementary Material Fig. 1), but only MWCNT-7 could be detected in the pleural cavity lavage cell pellet and extra-pulmonary organs (see Supplementary Material Fig. 1, 2). This suggests that the DWCNT fibers that did not become entrapped in granulation tissue could be transported into extra-pulmonary sites via the lymphatic system, but that these fibers were readily cleared from extra-pulmonary tissue, indicating low extra-pulmonary biopersistence.

One consequence of the translocation of MWCNT-7 fibers to the pleura is potential interaction of fibers with the mesothelium and pleural phagocytes. Such an interaction could result in tissue damage accompanied by tissue repair and generation of reactive oxidants. In MWCNT-7-treated rats, total protein was increased in the pleural cavity lavage fluid and the PCNA index of the visceral mesothelium was increased, suggesting fiber-associated tissue damage and repair did occur in the pleura of MWCNT-7-treated rats. Neither of these parameters was increased in DWCNT-treated rats.

Carcinogenesis requires that DNA damage results in fixation of mutations in the genome. Given the extremely high fidelity of the DNA monitoring and repair systems of mammalian cells, especially in long-lived species such as humans, the most likely route by which DNA damage subsequently results in mutation is damage of the DNA of replicating cells: damaged DNA in replicating cells is less likely to be repaired before the DNA is replicated than damaged DNA in resting cells. Chemically stable fibers that have aerodynamic diameters that allow deposition beyond the ciliated airways in the lung and that are of sufficient length and stability to make them biopersistent can cause damage to the DNA of replicating cells. Through interactions with macrophages and tissue cells, these fibers induce an inflammatory response and the production of reactive oxidants (Donaldson et al. 2011). These responses in turn cause tissue damage and subsequent tissue repair. Consequently, tissue repair occurs in the presence of DNA-damaging oxidants, resulting in damage to the DNA of replicating cells. Similar to asbestos fibers, MWCNT-7 and MWCNT-N, two long, thin, chemically stable CNTs, have been shown to be carcinogenic in rats after exposure via inhalation to MWCNT-7 (Kasai et al. 2016) or by TIPS to MWCNT-N (Suzui et al. 2016). In the present short-term study, inflammatory changes were milder in rats administered DWCNT compared to rats administered MWCNT-7, and parameters related to tissue damage (total protein, LDH levels, and ALP levels), DNA damage (8-OHdG adduct formation), and tissue repair (elevated PCNA index) were elevated in rats treated with MWCNT-7, but none of these parameters were elevated in rats treated with DWCNT (see Tables 1, 2; see Supplementary Material Doc 1 for a more detailed discussion of how these parameters relate to possible carcinogenicity).

In inhalation experiments, rats can exhibit very low sensitivity to respirable carcinogens. The crocidolite asbestos fiber burden required for a carcinogenic response in rats is approximately 1250 fibers/µg dry lung tissue while the fiber burden in humans that can lead to increased tumor risk is approximately 0.2 fibers/µg dry lung weight (Rodelsperger and Woitowitz 1995; also see; Bignon et al. 1995, p. 100). This 6000-fold difference in sensitivty to crocidolite asbestos led Rödelsperger and Woitowitz to conclude that “inhalation studies in rats are not sufficiently sensitive for the detection of hazards and risks to humans exposed to man-made fibres”. In another carcinogenicity study, Mohr et al. (2006) state: “moreover, the relatively low sensitivity of the inhalation model does not provide good conditions for producing dose–response relationships of different dust samples, which are necessary for analyses of best associations between certain dust properties and tumor responses. Therefore, the instillation model was used to investigate such relationships—independent of the fact that only an instillation study was realizable because of its much lower costs”. Administration of a test material via the airway using TIPS results in the highest concentrations of the test material in the lung at the beginning of the study period, allowing processes involved in carcinogenesis to proceed for the entire experimental period. In contrast, administration by inhalation results in the highest concentrations of the test material in the lung at the end of the experimental period. This pattern can contribute to higher sensitivity to respirable carcinogens administered by TIPS when the carcinogenic process is prolonged, such as development of mesotheliomas (see Fukushima et al. 2018). For example, administration of MWCNT-N (a long, thin CNT) via TIPS resulted in development of both lung tumors and mesotheliomas (Suzui et al. 2016), while administration of MWCNT-7 (another long-thin CNT) by whole-body inhalation resulted in the development of lung tumors but not mesotheliomas (Kasai et al. 2016). However, rats exposed to MWCNT-7 did accumulate fibers in the pleural cavity and did develop mesothelial hyperplasia, and if exposure to MWCNT-7 could have been continued for a further 1 or 2 years, fibers would have continued to accumulate in the pleural cavity and it is possible that the hyperplastic lesions would have developed into mesotheliomas.

In summary, we administered DWCNT and MWCNT-7 to rats using TIPS. The results of our initial short-term study indicate that compared to MWCNT-7, DWCNT induces a low degree of pulmonary toxicity and no discernable pleural toxicity, and the carcinogenic potential of DWCNT is likely to be much lower than that of MWCNT-7. The primary lesion caused by administration of DWCNT to the rat lung was the formation of granulation tissue with internalized DWCNT. The long-term consequences of these lesions and of other possible effects of DWCNT in the rat lung are being investigated in our ongoing 2-year study.

## Electronic supplementary material

Below is the link to the electronic supplementary material.

Supplementary material 1 (PDF 5152 KB)

Supplementary material 2 (PDF 12818 KB)
