# Supplementary material for: Comparative pulmonary toxicity of a DWCNT and MWCNT-7 in rats
Source: Arch Toxicol. 2018 Oct 19;93(1):49–59. doi: 10.1007/s00204-018-2336-3 (PMC6343020; doi:10.1007/s00204-018-2336-3)
Supplement: Supplementary file 2 — Supplementary material 2 (PDF 12818 KB) [file 204_2018_2336_MOESM2_ESM.pdf]

Archives of Toxicology

Comparative Pulmonary Toxicity of DWCNT and MWCNT-7 in Rats

Ahmed M. El-Gazzar[1,2,6], Mohamed Abdelgied[1,2,8], David B. Alexander[1], William T. Alexander[1], Takamasa Numano[1], Masaaki Iigo[1], Aya Naiki[2], Satoru Takahashi[2], Hiroshi Takase[3], Akihiko Hirose[4], Jun Kannno[5], Osama Saeid Elokle[6], Ashraf Mohamed Nazem[7], and Hiroyuki Tsuda[1]

[1]Nanotoxicology Project, Nagoya City University, Nagoya, Japan.

[2]Dept Exp Pathology and Tumor Biology, Nagoya City University Grad School Med Sciences, Nagoya, Japan.

[3]Core Laboratory Nagoya City University Graduate School Med Sciences, Nagoya, Japan.

[4]Div Risk Assessment, National Institute of Hygienic Sciences, Kawasaki, Japan.

[5]Japan Industrial Safety and Health Association, Japan Bioassay Research Center, Kanagawa, Japan.

[6]Dept Forensic Medicine and Toxicology, Faculty of Veterinary Medicine, Alexandria University, Alexandria, Egypt.

[7]Dept Forensic Medicine and Toxicology, Faculty of Veterinary Medicine, Beni Suef University, Beni Suef, Egypt.

[8]Dept Food Hygiene, Faculty of Veterinary Medicine, Alexandria University, Alexandria, Egypt.

Correspondence:
Hiroyuki Tsuda and David B. Alexander,
Nanotoxicology Project, Nagoya City
University, Nagoya, Japan.
Emails: htsuda@phar.nagoya-cu.ac.jp (H.T.),
dalexand@phar.nagoya-cu.ac.jp (D.B.A.)
ORCID 0000-0002-9017-3639 (D.B.A.)

Supplementary Table 1: Sequences of the actin, CCL2, CCL3, and CCL4 primers used for real time PCR.

Gene	Forward	Reverse
**Actin**	GGGAAATCGTGCGTGACA	AGGAAGGAAGGCTGGAAGA
**CCL2**	GCTACTCATTCACTGGCAAGAT	CTCTGTCATACTGGTCACTTCTAC
**CCL3**	CGACTGCCTGCTGCTTCT	TTCCTTGCTGCCTCTAATCTCA
**CCL4**	CTCTGCGTGTCTGCCTTCT	GCTGCTGGTCTCATAGTAATCC

Supplementary Table 2: Relative RNA expression at week 8.

	**Untreated**	**Vehicle**[a]	**DWCNT 0.25 mg**	**DWCNT 0.50 mg**	**MWCNT-7 0.25 mg**	**MWCNT-7 0.50 mg**
**CCL2**	0.27 ± 0.08	0.26 ± 0.14	1.16 ± 0.34**	1.28 ± 0.31***	1.65 ± 0.61***	2.42 ± 0.64***,###
**CCL3**	6.40 ± 2.23	7.29 ± 2.54	10.66 ± 1.54	14.43 ± 2.50**	16.50 ± 5.79***,#	18.03 ± 3.87***
**CCL4**	3.25 ± 0.49	3.91 ± 1.16	3.63 ± 0.69	4.25 ± 1.24	8.49 ± 2.43***,###	9.38 ± 2.76***,###

[a] There were no significant differences between the Untreated group and the Vehicle Control group.
,* Different from the Vehicle Control at p<0.01, p<0.001, respectively.
#,### Different from the respective DWCNT group at p<0.05, p<0.001, respectively.

Supplementary Table 3: Pulmonary macrophage counts, PLF total protein and LDH levels, and PCNA indexes at week 3.

	**Untreated**	**Vehicle**[a]	**DWCNT 0.25 mg**	**DWCNT 0.50 mg**	**MWCNT-7 0.25 mg**	**MWCNT-7 0.50 mg**
**Pulmonary Macrophage Count per cm^2 (x 10^3)**	4.65 ± 0.97	12.38 ± 1.47	13.19 ± 2.27	12.70 ± 0.97	19.86 ± 3.34***,###	19.40 ± 2.88***,###
**PLF - Total Protein (µg/ml)**	757 ± 24	758 ± 34	755 ± 31	810 ± 32	813 ± 71	920 ± 118***,#
**PLF - LDH (mU/mL)**	4.08 ± 1.47	5.83 ± 2.33	6.44 ± 4.10	5.24 ± 2.31	7.20 ± 4.71	11.97 ± 5.85*,#
**Pulmonary PCNA Index (%)**	2.2 ± 0.8	3.9 ± 1.1	6.8 ± 1.6	11.3 ± 2.0***	14.3 ± 3.9***,###	14.8 ± 4.3***
**Visceral Pleura PCNA Index (%)**	5.5 ± 2.4	7.6 ± 2.3	3.3 ± 2.7	7.1 ± 2.2	11.5 ± 4.3	10.3 ± 2.5

[a] Macrophage count was significantly higher in the vehicle control group compared to the untreated control group (p < 0.001)
*,*** Different from the Vehicle Control at p<0.05, p<0.001, respectively.
#,### Different from the respective DWCNT group at p<0.05, p<0.001, respectively.

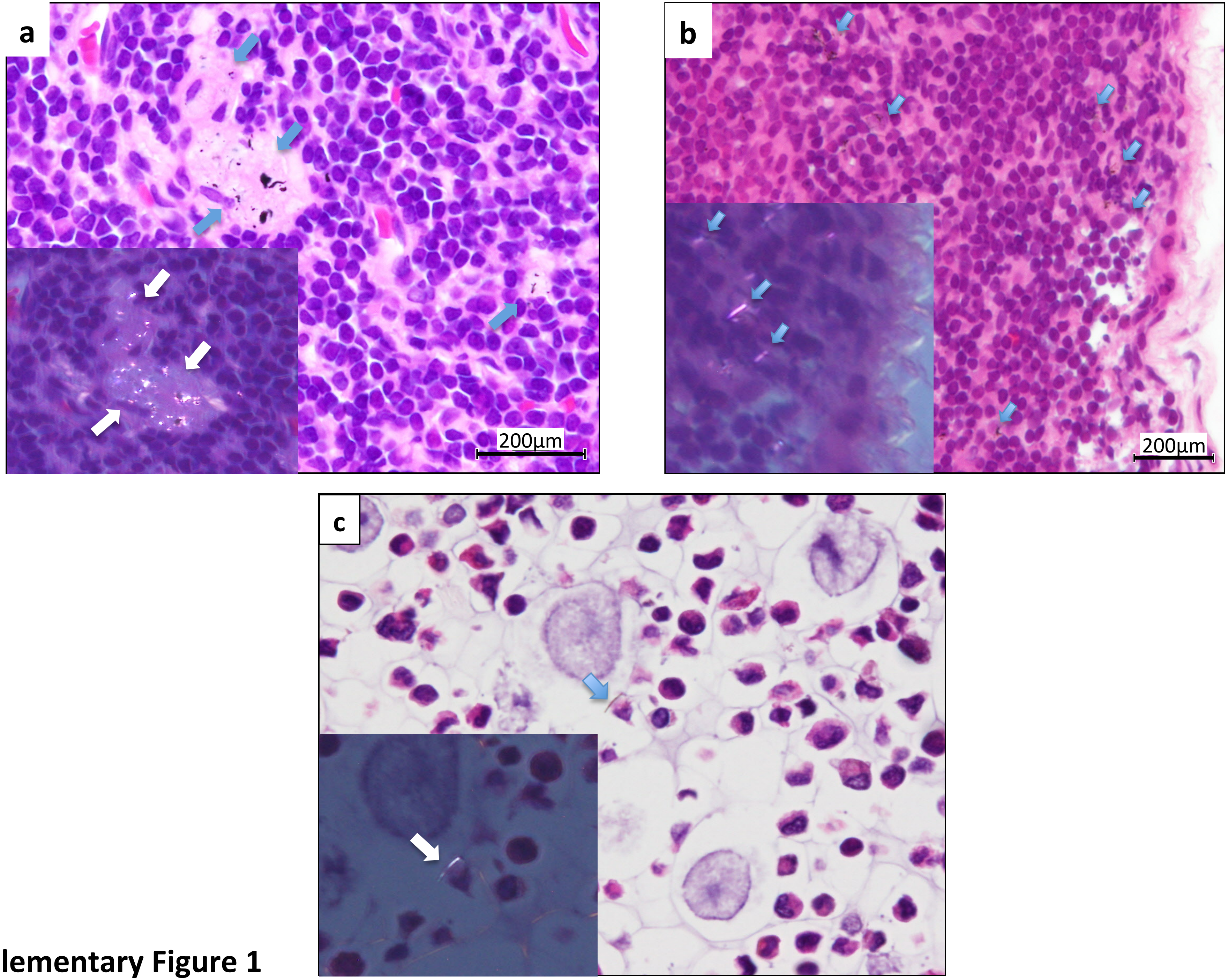

Supplementary Figure 1

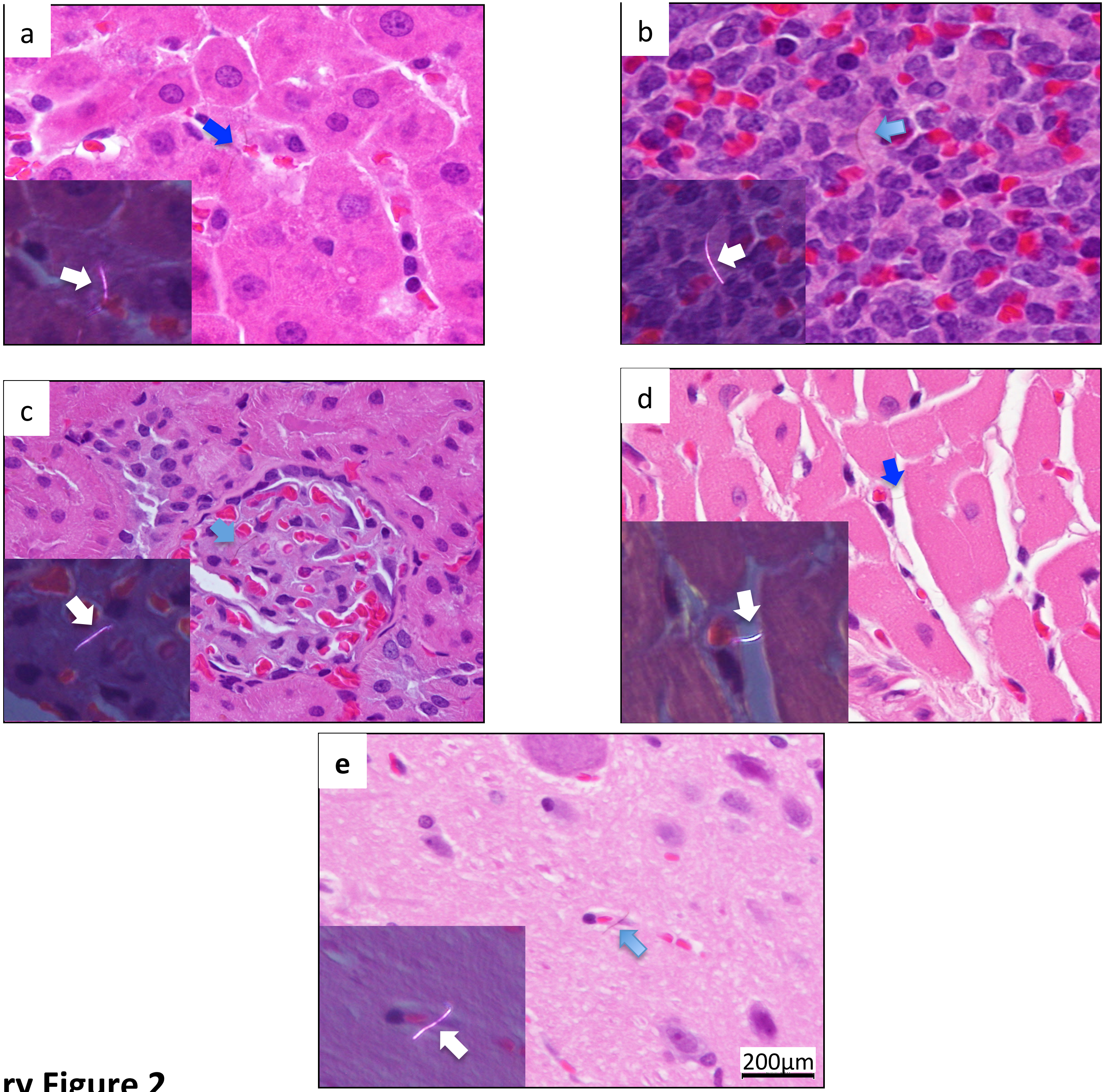

Supplementary Figure 2

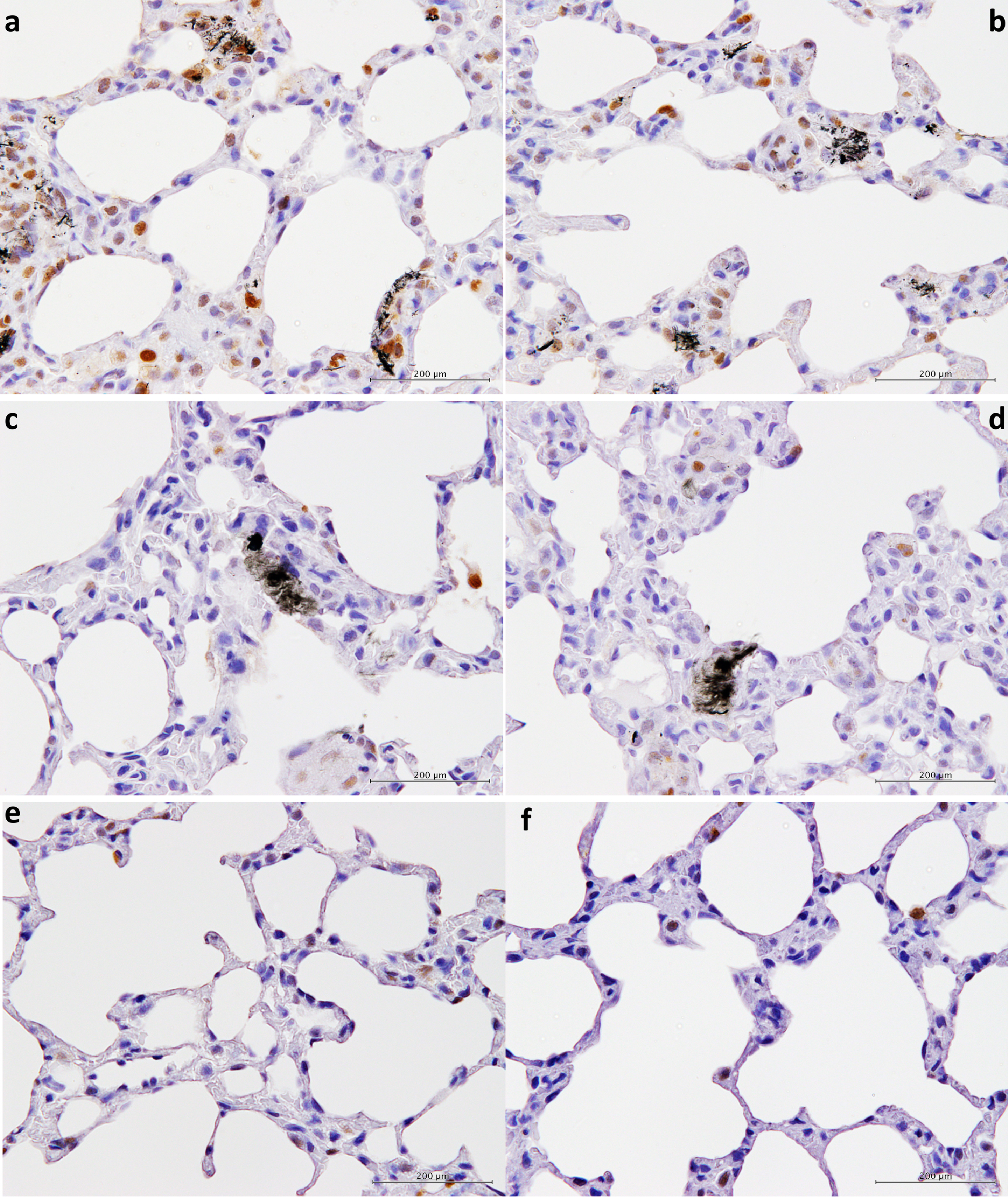

Supplementary Figure 3

Supplementary Figure Legends

Supplementary Figure 1 Translocation of the DWCNT fibers (a) and MWCNT-7 fibers (b) to the mediastinal lymph node, and the presence of MWCNT-7 fibers (c) in the pleural cavity lavage cell pellet at week 6. Large swollen cells are degenerating macrophages. DWCNT fibers were not found in the pleural cavity lavage cell pellet.

Supplementary Figure 2 Translocation of MWCNT-7 fibers to the liver sinusoid (a), lymphatic sheath of the spleen (b), kidney glomerulus (c), heart stoma (d), and brain (e) at week 6. DWCNT fibers were not found in any extrapulmonary organs.

Supplementary Figure 3 Proliferating cell nuclear antigen (PCNA) staining of the lung at week 8. (A) In rats administered 0.50 mg MWCNT-7, fibers were dispersed throughout the lungs. PCNA positive pulmonary epithelial cells and macrophages were associated with these fibers. The PCNA index in the lungs of these rats was 11.8. (B) In rats administered 0.25 mg MWCNT-7, fibers were also dispersed throughout the lungs. Similarly to the 0.50 mg MWCNT-7 group, PCNA positive pulmonary epithelial cells and macrophages were associated with these fibers. The PCNA index in the lungs of these rats was 9.9. (C) Fibers were also distributed throughout the lungs of rats administered 0.50 mg DWCNT. However, there were fewer PCNA positive pulmonary epithelial cells and macrophages were associated with these fibers than with the MWCNT-7 fibers. The PCNA index in the lungs of these rats was 5.4. (D) Similarly to the 0.50 mg DWCNT group, fibers were distributed throughout the lungs of rats administered 0.25 mg DWCNT, and again, like the 0.50 mg DWCNT group, there were fewer PCNA positive pulmonary epithelial cells and macrophages associated with these fibers than with the MWCNT-7 fibers. The PCNA index in the lungs of these rats was 5.8. (E) The PCNA index in the lungs of the rats treated with vehicle alone was 3.3. (F) The PCNA index in the lungs of the untreated rats was 3.1.
